# Real-Life Use of [68Ga]Ga-DOTANOC PET/CT in Confirmed and Suspected NETs from a Prospective 5-Year Electronic Archive at an ENETS Center of Excellence: More Than 2000 Scans in More Than 1500 Patients

**DOI:** 10.3390/cancers16040701

**Published:** 2024-02-07

**Authors:** Norma Bonazzi, Emilia Fortunati, Lucia Zanoni, Giulia Argalia, Diletta Calabrò, Elena Tabacchi, Vincenzo Allegri, Davide Campana, Elisa Andrini, Giuseppe Lamberti, Martina Di Franco, Riccardo Casadei, Claudio Ricci, Cristina Mosconi, Stefano Fanti, Valentina Ambrosini

**Affiliations:** 1 Nuclear Medicine, Alma Mater Studiorum, University of Bologna, 40126 Bologna, Italy; argalia.giulia@gmail.com (G.A.); dilecala@gmail.com (D.C.); martina.difranco2@studio.unibo.it (M.D.F.); stefano.fanti@aosp.bo.it (S.F.); valentina.ambrosini@unibo.it (V.A.); 2 Nuclear Medicine, IRCCS, Azienda Ospedaliero-Universitaria di Bologna, 40138 Bologna, Italy; emilia.fortunati@studio.unibo.it (E.F.); lucia.zanoni@aosp.bo.it (L.Z.); elena.tabacchi@aosp.bo.it (E.T.); vincenzo.allegri@aosp.bo.it (V.A.); 3 Medical Oncology, IRCCS, Azienda Ospedaliero-Universitaria di Bologna, 40138 Bologna, Italy; davide.campana@unibo.it (D.C.); elisa.andrini3@unibo.it (E.A.); giuseppe.lamberti8@unibo.it (G.L.); 4 Department of Medical and Surgical Sciences (DIMEC), Alma Mater Studiorum, University of Bologna, 40126 Bologna, Italy; cristina.mosconi@aosp.bo.it; 5 Department of Internal Medicine and Surgery (DIMEC), Alma Mater Studiorum, University of Bologna, 40126 Bologna, Italy; riccardo.casadei@unibo.it (R.C.); claudio.ricci6@unibo.it (C.R.); 6 Division of Pancreatic Surgery, IRCCS, Azienda Ospedaliero-Universitaria di Bologna, 40138 Bologna, Italy; 7 Department of Radiology, IRCCS, Azienda Ospedaliero-Universitaria di Bologna, 40138 Bologna, Italy

**Keywords:** PET/CT, [68Ga]Ga-DOTANOC, neuroendocrine neoplasms, neuroendocrine tumors, indications

## Abstract

**Simple Summary:**

Neuroendocrine neoplasms’ (NENs) rarity and heterogeneity represent a clinical challenge. Somatostatin receptor (SST) positron emission tomography/computed tomography (PET/CT) availability and different reimbursement policies across countries account for its heterogenous employment on a single-case basis. The aim of this study was to prospectively collect data of the real-life use of and indications for [68Ga-DOTA^0^-1NaI^3^]octreotide ([68Ga]Ga-DOTANOC) PET/CT in a prospective 5-year electronic archive at a single center. Overall, more than 2000 scans were included. This systematic data collection in a high-volume diagnostic center represents a reliable cohort reflecting the trends of [68Ga]Ga-DOTANOC PET/CT use across different clinical indications and primary tumor sites.

**Abstract:**

The recent introduction of novel treatments for advanced neuroendocrine tumors (NETs) and the well-established impact of clinical case discussion within dedicated multidisciplinary teams indicates the need to promote the centralization of rare diseases, such as NENs (neuroendocrine neoplasms). Data on the real-life use of and indications for [68Ga]Ga-DOTANOC PET/CT were collected from a prospective monocentric 5-year electronic archive including consecutive patients with confirmed and suspected NETs (September 2017 to May 2022). Overall, 2082 [68Ga]Ga-DOTANOC PET/CT scans (1685 confirmed NETs, 397 suspected NETs) were performed in 1537 patients. A high positivity rate was observed across different clinical settings (approximately 70%). Approximately 910/2082 scans were requested by the local oncology ward (851 confirmed NETs, 59 suspected NETs). The following observations were found: (i) the detection rate across all indications was 73.2% (higher for staging, peptide receptor radioligand therapy (PRRT) selection, and treatment response assessment); (ii) in suspected NETs, PET was more often positive when based on radiological findings. This systematic data collection in a high-volume diagnostic center represents a reliable cohort reflecting the global trends in the use of [68Ga]Ga-DOTANOC PET/CT for different clinical indications and primary tumor sites, but prompts the need for further multicenter data sharing in such a rare and slowly progressive disease setting.

## 1. Introduction

PET/CT plays a pivotal role in neuroendocrine neoplasm (NEN) diagnosis; in particular -[68Ga]Ga-DOTA peptides (TOC, TATE, and NOC) are routinely used to image well-differentiated neuroendocrine tumors (NETs) at different timepoints of diseases’ natural course. 

NENs are a group of rare heterogenous tumors with variable primary tumor sites, clinical presentation, and behavior overtime [1,2,3]. NENs are mostly slow-growing and non-functioning, originating from the secretory cells of the neuroendocrine system, widely dispersed in the human body. NENs share pathological characteristics and markers; they are outlined by cell origin, differentiation, and proliferation rate [4]. The most recent (2022) update of the WHO classification of endocrine and neuroendocrine tumors [4] categorized NENs, depending on the cell of origin, as tumors of neural type and tumors of epithelial type. Epithelial NENs received further classification based on the cells’ differentiation grade: NETs, if well-differentiated, and neuroendocrine carcinomas (NEC), if poorly differentiated [4]. NETs are further categorized on the basis of Ki67: G1 (Ki-67 < 3%), G2 (Ki-67 3–20%), and G3 (Ki-67 > 20%). NEC, conversely, are intrinsically high-grade and show a high proliferation rate (Ki-67 is typically even higher than 55%) [4]. This grading system correlates with prognosis, with higher grades showing the poorest outcome. The majority of NETs (72%) arise from the gastro-entero-pancreatic (GEP) tract, followed by the bronchopulmonary system (25%), while other primary sites are less frequent (e.g., adrenals, thyroid, breast, prostate, and skin) [4]. A minority of cases present as inherited syndromes (e.g., von Hippel–Lindau disease, multiple endocrine neoplasia (MEN), neurofibromatosis, and tuberous sclerosis) [5]. 

The choice of the PET/CT radiopharmaceutical depends on pathological features. A hallmark characteristic of NETs is the expression of somatostatin receptors (SST) [4] that represent the target of SST radiopharmaceuticals (e.g., [68Ga-DOTA^0^-Tyr^3^]octreotate -[68Ga]Ga-DOTATATE-, [68Ga-DOTA^0^-Tyr^3^]octreotide -[68Ga]Ga-DOTATOC-, and [68Ga-DOTA^0^-1NaI^3^]octreotide -[68Ga]Ga-DOTANOC-) clinically employed for positron emission tomography (PET) imaging [6,7,8]. Different radiopharmaceuticals are preferentially employed for NETs with variable or low SST expression (e.g., L-6-[18F]fluoro-3,4-dihydroxyphenylalanine-[18F]DOPA- for medullary thyroid carcinoma) or undifferentiated forms (e.g., 2-deoxy-2-[18F]fluoro-D-glucose-[18F]FDG- for high-grade G2 and G3 NETs and NEC) [9,10,11,12].

With the approval of [177Lu][Lu-DOTA^0^-Tyr^3^]octreotate ([177Lu]Lu-DOTATATE) radioligand therapy [13] as a second-line treatment for patients with metastatic progressive disease on cold somatostatin analogues, in vivo assessment of SST status is crucial for the selection of patients eligible for PRRT (peptide receptor radioligand therapy) [9,14,15].

According to the European Association of Nuclear Medicine (EANM) guidelines, current PET/CT (positron emission tomography/computed tomography) indications include baseline staging (at the time of diagnosis and prior to surgical resection), restaging (including cases of suspected relapse based on both clinical/laboratory or imaging findings), response to therapy assessment, evaluation of PRRT eligibility, identification of the unknown primary tumor site (CUP, cancer of unknown primary), and follow-up and evaluation of suspected NETs [9,16].

Considering the disease rarity and heterogeneity, NENs represent a challenge for the clinician. Different SST PET/CT availability among centers and variable reimbursement policies across countries also account for PET/CT heterogenous employment in NET management on a single-case basis.

Our hospital is part of the European Reference Network (ERN) and was accredited as a European Neuroendocrine Tumor Society (ENETS) center of excellence and locally promoted NEN patients’ multidisciplinary management (with different professional expertise and advanced diagnostic procedures, all locally available). Our local scenario, including patients referred from the whole country, represents an optimal setting where data (e.g., demographical, surgical, clinical, laboratory, imaging, and pathological) can be systematically collected to assess the real-life use of SST PET/CT in NET management at different time points of the clinical presentation.

Therefore, the aim of this paper was to analyze data prospectively collected from a 5-year electronic archive of [68Ga]Ga-DOTANOC PET/CT scans requested for confirmed or suspected NETs to observe PET/CT real-life use and indications.

## 2. Materials and Methods

Clinical and imaging data of consecutive patients with pathological diagnosis or suspicion of NETs referred for [68Ga]Ga-DOTANOC PET/CT at our center were collected in a 5-years prospective electronic archive (September 2017 to May 2022; EC number 131/2017/O/Oss) designed on a scan basis. Patients signed an informed consent form before each PET/CT scan. A subgroup of the included scans was requested by the local oncology unit (EC number 626/2020/Oss/AOUBo).

Prospectively collected data included detailed clinical history (e.g., primary tumor site, WHO grade, previous or on-going treatment, and comorbidities) and the results of other radiological and functional imaging, when available.

[68Ga]Ga-DOTANOC PET/CT was performed according to the standard EANM procedure [10] (injected dose: 100–200 MBq; uptake time: 60–90 min after injection; field of view: from the skull base to the mid-thigh; no specific preparation). [68Ga]Ga-DOTANOC was synthesized onsite [17,18] at our local radiopharmacy. Areas of increased radiopharmaceutical uptake outside the normal biodistribution sites (e.g., spleen/accessory spleens, kidneys/bladder, uncinate process/head of the pancreas, adrenals, pituitary, thyroid, and liver) were considered pathological. Exams were reviewed by experienced nuclear medicine physicians aware of potential pitfalls of [68Ga]Ga-DOTANOC imaging (e.g., non-neuroendocrine tumors expressing somatostatin receptors; infectious or inflammatory findings). Nuclear medicine physicians conducting the PET/CT at our center have a long-standing experience with [68Ga]Ga-DOTANOC PET/CT reporting (started in 2007) based on a high volume of scans performed each year.

Analysis was therefore performed on a scan basis. Positivity rate (ratio of number of positive scans to total number of scans) was calculated. In the subgroup of scans requested by the local oncology ward, detection rates (ratio of number of true positive scans to total number of scans) were also assessed using corresponding clinical and imaging results to validate PET findings. Descriptive analysis was performed, and all categorical variables were reported as frequencies and percentages.

## 3. Results

### 3.1. Whole Population

Overall, during the 5-year timeframe, 2082 [68Ga]Ga-DOTANOC-PET/CT scans were performed in 1537 patients (304 out of 1537 patients had double or more [68Ga]Ga-DOTANOC-PET/CT). Analysis was therefore performed on a scan basis, according to the electronic archive design. [68Ga]Ga-DOTANOC-PET/CT results were positive in 1326/2082 scans (63.7%) and negative in 756/2082 (36.3%). In particular, PET/CT results were positive in 186/254 (73.2%) of pre-treatment staging scans, in 103/249 (41.4%) of post-surgical scans, in 34/36 (94.4%) scans evaluated for PRRT eligibility, in 277/299 (92.6%) assessed during treatment, in 88/99 (88.9%) restaged after therapy, in 127/195 (65.1%) of the suspected relapse subgroup, in 284/479 (59.3%) studied during follow-up, in 57/74 (77.0%) of CUP scans, and in 170/397 (44.9%) of suspected NET scans.

Disease confined to the primary tumor site was detected in 437/1326 (33%), while metastatic disease was observed in 889/1326 (67%) scans. Most common metastatic sites were nodes and the liver, followed by bone and the lungs; nodal-only involvement was observed in 227/889 scans (25.5%), while diffuse metastatic spread was found in 662/889 scans (74.5%), with or without nodal involvement. The most common site of metastatic spread was the liver, either alone (193/889, 21.7%) or with associated nodal disease (145/889; 16.3%). Bone-only metastatic spread was detected in 32/889 (3.6%) and lungs-only in 16/889 (1.8%). Various associations of different metastatic sites were observed in the remaining scans.

Additional PET non-malignant findings were common (1038/2082 scans; 49.9%): pancreatic head/uncinate process physiological uptake (571/1038, 55.0%), accessory spleens (49/1038, 4.7%), thyroid (160/1038, 15.4%), prostate (182/1038, 17.5%), lungs (inflammatory/infectious; 53/1038, 14.7%), bone (31/1038, 3.0%), brain (31/1038, 3.0%; of which 26/31 were incidental meningiomas), nodes (66/1038, 6.4%), uterine (49/1038, 4.7%), ureters (19/1038, 1.8%), adrenal glands (20/1038, 1.9%), stomach (42/1038, 4.0%), bowel (84/1038, 8.1%), pituitary gland (12/1038, 1.2%), liver (28/1038, 2.7%), ovaries (1/1038, 0.1%), thymus (2/1038, 0.2%), parathyroids (1/1038, 0.1%), breasts (14/1038, 1.3%), tonsils (1/1038, 0.1%), salivary glands (1/1038; parotid gland, 0.1%), and miscellaneous known inflammatory/infectious processes (24/1038, 2.3%).

#### 3.1.1. Pathologically Confirmed NETs

On a scan-based analysis, 1685 scans (1685/2082, 80.9%) were performed in pathologically confirmed NETs, while 397 scans were performed in suspected NETs. 

**Figure 1:**
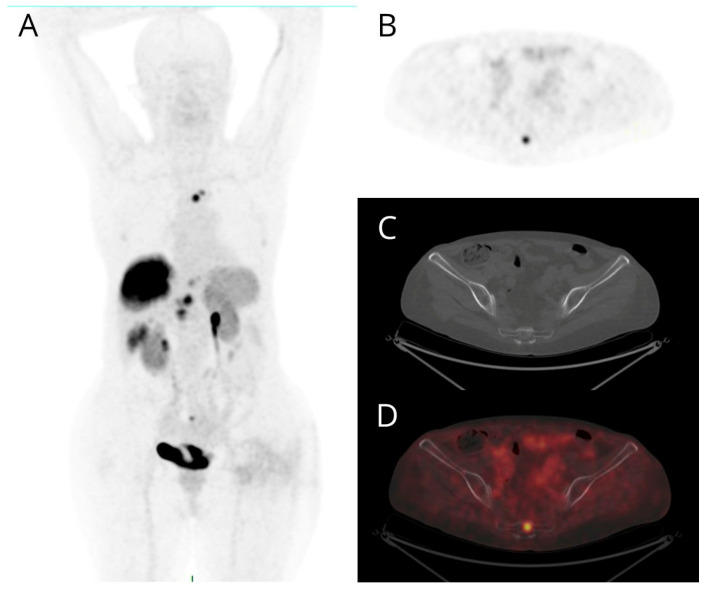
Maximum intensity projection (MIP) (**A**), PET (**B**), non-diagnostic CT (**C**), and fused PET/CT (**D**) transaxial images of a patient with metastatic G2 pancreatic NET. Bone lesions exhibit intense [68Ga]Ga-DOTANOC uptake (**B**,**D**) without morphologically evident corresponding lesion on non-diagnostic CT (**C**). The other PET-positive findings (**A**) are two pancreatic nodules, a voluminous hepatic lesion, and further smaller lesions in the VI segment and in the left lobe of the liver, a bone lesion of the sternum and a mediastinal node.

Most scans were performed in GEP NETs (1135/1685, 67.4%. Table 1), with the pancreas (Figure 1) and ileum being the most frequently studied, and G1 and G2 the most common grades (Table 2). Scans performed in lung NETs were 188/1685 (11.2%), with a majority of typical carcinoids (101/188, 53.7%). 

Most scans were performed for staging (503/1685, 29.9%), treatment assessment (398/1685, 23.6%), suspected relapse (195/1685, 11.6%), follow-up (479/1685, 28.4%), or CUP (74/1685, 4.4%) (see detailed data for all indications in Table 1). Overall, PET/CT results were positive in 1156/1685 scans in confirmed NETs (68.6%; Table 1).

#### 3.1.2. Suspected NETs

**Figure 2:**
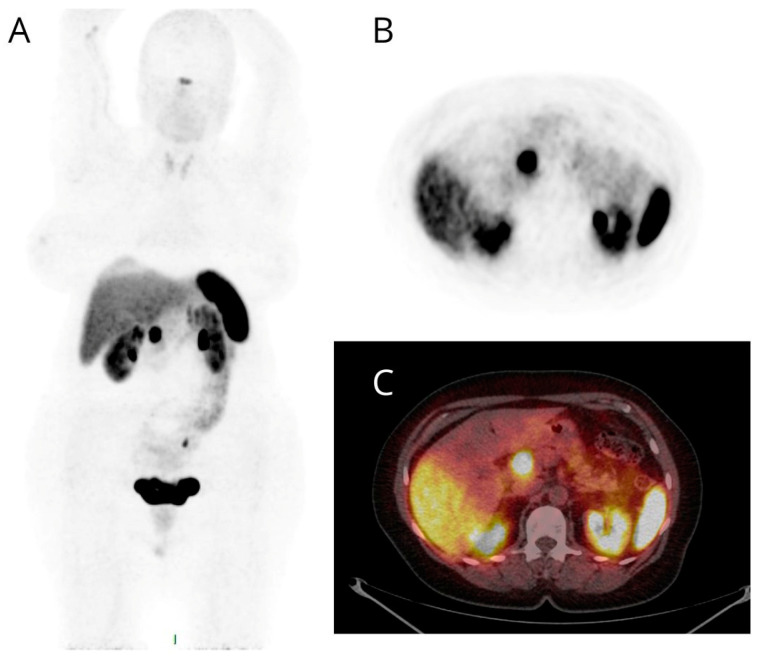
Maximum intensity projection (MIP) (**A**), PET (**B**), and fused PET/CT (**C**) transaxial images of a patient studied for suspected NET based on diagnostic contrast-enhanced CT detection of a nodule of the pancreatic head. High and focal [68Ga]Ga-DOTANOC uptake (**B**,**C**) was evident at the pancreatic head and later confirmed by pathological assessment.

Overall, 397 scans were performed in suspected NETs (without histological confirmation) and resulted more frequently in being positive when the suspicion was based on radiological (151/306, 49.3%) (Figure 2) rather than clinical/laboratory findings (19/91, 20.9%).

#### 3.1.3. Subgroup Analysis of Scans (n = 851) Performed in Confirmed NETs, Requested by the Local Oncology Ward

Most scans (Table 3) were performed in GEP NETs (633/851, 74.4%) and lung carcinoids (75/851, 8.8%), with detection rates for SST-expressing lesions above 70%.

PET/CT scans were performed mostly for staging (overall, 204/851, 24%; staging before surgery: 77/204, 37.7%; staging after surgery: 127/204, 62.3%), therapy response assessment (overall, 224/851, 26.3%; interim: 172/224, 76.8%; post therapy: 52/224, 23.2%), and follow-up (282/851, 33.1%) (Table 3). Overall, PET/CT was positive in 623/851 scans (73.2%).

When scans were classified by primary tumor site in GEP NETs (Table 4), they were mostly performed in pancreas (37.8%) and ileum (51.5%) NETs, with an overall detection rate of 73.9%. The overall detection rate in scans performed for lung NETs was 70.7% (75% were typical carcinoids).

#### 3.1.4. Subgroup Analysis of Scans in Suspected NETs (n = 59), Requested by the Local Oncology Ward

In the subset of scans performed in suspected NETs (without pathological confirmation), the suspicion was based on radiological findings in 55/59 scans (93.2%) and on clinical/laboratory data in the remaining 4/59 (6.8%). Overall, PET/CT results were positive in 50/59 scans (84.7%), with a higher positivity rate when the suspicion was based on radiological findings (47/55, 85.5%). When the suspicion of NETs was based on radiological findings, 39/55 scans were later confirmed as true positives (37/39 well-differentiated, 94.9%; 2/39 atypical carcinoids, 5.1%), resulting in a global PET/CT detection rate of 70.9%. When the suspicion of NETs was based only on clinical/laboratory findings, only half of the scans (2/4) were later confirmed as true positives. Diagnostic confirmation of positive PET findings was not available in the remaining 16/55 scans with suspected NETs based on radiological findings and in 2/4 of the clinically suspected NETs.

## 4. Discussion

Although generally described as rare tumors in terms of incidence, it is well established that the prevalence of NENs is increasing due to improvements in imaging techniques, a deeper clinical awareness, and the introduction of novel and efficient treatment strategies [19]. However, our understanding of the disease is based mostly on relatively small cohort studies, often managed across different centers. Therefore, the collection and analysis of clinical and imaging data in a large patient population, with suspected or confirmed NETs, referred for functional imaging at the same center may allow us to draw a picture of the real-life use of [68Ga]Ga-DOTANOC PET/CT.

In a 5-year timeframe, 2082 [68Ga]Ga-DOTANOC PET/CT scans were performed in 1537 patients; overall, 1685 scans were performed in confirmed NETs, while 397 scans were performed in suspected NET cases. Most scans were performed in GEP (mainly pancreas, ileum, and duodenum) or lung NETs (mostly typical carcinoids), in line with the literature data [20,21,22], thus representing a reliable cohort reflecting the global NET trends. Less frequently, scans were performed for less common primary tumors (e.g., pheochromocytoma, paraganglioma, medullary thyroid cancer, insulinoma, meningioma, neuroblastoma, and NETs derived from the ear, breast, ovary, thymus, and salivary glands) [10]. It is to be noted that in our high-volume center, other radiopharmaceuticals are also available, such as [18F]FDG employed for high-grade tumors and [18F]DOPA for pheochromocytoma, neuroblastoma, and medullary thyroid cancer [10,23]. Only a minority of scans were performed in MEN [20]. 

The most common indications for [68Ga]Ga-DOTANOC PET/CT scanning were staging (both before and after surgery), therapy assessment, follow-up, and suspected NET, in line with current guidelines [10,24,25]. Less common indications included PRRT selection, mainly requested by the local oncology ward (22/36). This low percentage in the overall cohort was likely biased by data collection of patients not followed locally, in which the indication reported at the time of scanning was not specifically PRRT eligibility but rather restaging after therapy (indication for PRRT was likely taken subsequently to the PET/CT positive result). This indication is expected to consistently increase in the near future following the emerging demand for PRRT after the definitive approval of [177Lu]Lu-DOTATATE in Europe and in the United States [13,26]. 

When scans were classified by primary tumor site, the overall PET/CT positivity rate was approximately 68%. Additional PET non-malignant findings were observed in approximately half of the scans, including both well-known sites of para-physiological uptake/biodistribution (e.g., accessory spleens; pancreatic head/uncinate process) and inflammatory/infectious processes [10,27]. Comprehensive knowledge of potential pitfalls is crucial for accurate imaging interpretation and reporting.

Approximately half of the whole cohort included scans requested by the local oncology ward and PET/CT scan results could be validated by corresponding clinical and conventional imaging. The overall detection rate was 73.2%. It is important to note that the post-surgical detection rate was approximately half of the pre-surgical one (44.9% vs. 84.4%), likely due to the particular setting in which at least the primary tumor site was already resected. Pre-surgical SST PET/CT imaging [10] is recommended to both evaluate disease extent and to provide functional characterization of each lesion, as suggested by the European Society for Medical Oncology (ESMO), EANM, and ENETS guidelines [10,24,25]. 

PET/CT detection rate was above 90% either during or after treatment assessment, likely reflecting that most NET patients show stable disease or partial response to treatment [28].

Lower detection rates were observed in scans performed for follow-up, likely reflecting the generally slowly progressive disease and the need to further ameliorate the diagnostic surveillance protocols.

All of the indications for SST PET/CT imaging were in line with recently published appropriate use criteria [15] (e.g., initial staging after histologic diagnosis, the localization of primary tumor in patients with known metastatic disease but unknown primary, the selection of patients for SST-targeted PRRT, and the staging before planned surgery).

Among scans requested by the local oncology ward for suspected NETs, the detection rate was higher when the suspicion was based on conventional imaging findings as compared to clinical/laboratory findings. In this clinical setting, the pre-test probability of disease is certainly crucial to predict PET positivity: a multidisciplinary discussion is the best way to select the cases in which an SST PET/CT is really needed [29]. In fact, all scans requested by the local oncology ward were discussed in the NEN multidisciplinary meeting (where oncologists, surgeons, gastroenterologists, endocrinologists, radiologists, nuclear medicine physicians, and pathologists were all involved). The impact of a multidisciplinary approach is well known [26,30] and mandatory to optimize patients’ management. From a clinical perspective, the setting of suspected NETs is very complex and challenging: the EANM Focus 3 multidisciplinary discussion among experts reached consensus on the employment of both [68Ga]Ga-SST PET/CT and contrast-enhanced CT only after careful clinical assessment of pre-test probability of disease [31,32]. A particular setting is the presence of a lesion suspicious for NET but not amenable to bioptical sampling; here, SST PET/CT may provide useful data for management.

Limitations of the study are mostly represented by the heterogeneous data set including scans requested even by other centers (due to the high availability of PET/CT slots at our unit), for different primary tumors, and at various disease timepoints. This reflects also the lack of subsequent data when scans were performed in patients addressed elsewhere after the PET/CT result.

On the other hand, given the rarity and heterogeneity of NENs, a prospective electronic archive is essential to collect clinical and imaging data for better customization of the use of [68Ga]Ga-DOTANOC-PET/CT in the diagnostic flowchart. The recent introduction of novel treatment options for advanced NETs and the well-established impact of clinical case discussions within a dedicated multidisciplinary tumor board indicates the need to promote the centralization of rare diseases, such as NENs, and to improve multicenter data sharing to allow for longitudinal observation of a mostly slowly progressive disease.

## 5. Conclusions

Considering the observational nature of the present study, the value of the present paper is the focus on the routine employment of SST PET/CT across various indications and variable primary tumor sites. Real-life use of and indications for [68Ga]Ga-DOTANOC PET/CT were prospectively collected in a 5-year monocentric electronic archive (more than 2000 scans in more than 1500 patients). Most scans were performed in GEP and lung NETs but also in less common tumor sites; [68Ga]Ga-DOTANOC PET/CT showed a high positivity rate across all indications. Also, in the subgroup of PET/CT scans requested by the local oncology ward, most scans were performed in GEP and lung NETs. The detection rate was higher in scans performed for staging, PRRT selection, and treatment response assessment. The suspicion of NET was confirmed more often when based on radiological findings rather than when only based on clinical/laboratory findings.

## Figures and Tables

**Table 1:** A total of 1685 scans in confirmed NETs.

	n	%	PET/CT		
			pos	neg	Positivity Rate
Scans Classified by Primary Tumor Site			n	n	%
GEP	1135	67	785	350	69.2
Lung	188	11	111	77	59.0
Pheochromocytoma	13	1	9	4	69.2
Paraganglioma	26	2	21	5	80.8
Medullary thyroid cancer	14	1	11	3	78.6
Insulinoma	6	0	2	4	33.3
Meningioma	11	1	11	0	100.0
Ear	18	1	8	10	44.4
Breast	10	1	4	6	40.0
Ovary	4	0	3	1	75.0
Thymus	11	1	6	5	54.5
MEN	69	4	57	12	82.6
Neuroblastoma	1	0	1	0	100.0
Salivary glands	4	0	1	3	25.0
MEN + GIST	4	0	4	0	100.0
NA	171	10	122	49	71.3
Total	1685	100	1156	529	68.6
	**n**	**%**	**PET/CT**		
			**pos**	**neg**	**Positivity Rate**
**Scans Classified by Indication of PET/CT**			**n**	**n**	**%**
Staging	503	30	289	214	57.5
*Pre-surgical*	254		186	68	73.2
*Post-surgical*	249		103	146	41.4
PRRT selection	36	2	34	2	94.4
Treatment response assessment					
*Interim*	299	18	277	22	92.6
*Post-treatment*	99	6	88	11	88.9
Suspected relapse	195	12	127	68	65.1
Follow-up	479	28	284	195	59.3
Unknow primary tumor	74	4	57	17	77.0
Total	1685	100	1156	529	68.6

Legend: NET = neuroendocrine tumor, PET/CT = positron emission tomography/computed tomography, n = number, pos = positive, neg = negative, GEP = gastro-entero-pancreatic, MEN = multiple endocrine neoplasia, GIST = gastrointestinal stromal tumor, NA = not available, and PRRT = peptide receptor radioligand therapy.

**Table 2:** Scans performed in confirmed GEP NETs.

	n	%
Scans Classified by Primary Tumor Site		
Pancreas	419	36.9
Ileum	529	46.6
Pancreas and ileum	2	0.2
Duodenum	38	3.3
Jejunum	13	1.1
Colon	17	1.5
Sigma	6	0.5
Rectum	15	1.3
Stomach	41	3.6
Cecum	6	0.5
Vater’s papilla	5	0.4
Appendix	26	2.3
Gallbladder	5	0.4
Liver	4	0.4
Ileocecal valve	9	0.8
Total	1135	100.0
	**n**	**%**
**Scans Classified by Grading**		
G1	432	38.1
G2	499	44.0
G3	22	1.9
NET_grade not specified	33	2.9
NA	149	13.1
Total	1135	100.0

Legend: NET = neuroendocrine tumor, n = number, GEP = gastro-entero-pancreatic, and NA = not available.

**Table 3:** Scans performed in confirmed NETs, requested by the local oncology ward.

	n	PET/CT		
		pos	neg	Detection Rate
Scans Classified by Primary Tumor Site		n	n	%
GEP	633	468	165	73.9
Lung	75	53	22	70.7
Pheochromocytoma	3	2	1	66.7
Paraganglioma	1	0	1	0.0
Ear	7	4	3	57.1
Breast	8	2	6	25.0
Ovary	1	0	1	0.0
Thymus	2	0	2	0.0
MEN	53	43	10	81.1
Salivary glands	1	0	1	0.0
NA	67	51	16	76.1
Total	851	623	228	73.2
	**n**	**PET/CT**		
		**pos**	**neg**	**Detection Rate**
**Scans Classified by Indication of PET/CT**		**n**	**n**	**%**
Staging				
Pre-surgical	77	65	12	84.4
Post-surgical	127	57	70	44.9
PRRT selection	22	22	0	100.0
Treatment response assessment				
Interim	172	162	10	94.2
Post-treatment	52	48	4	92.3
Suspected relapse	86	63	23	73.3
Follow-up	282	179	103	63.5
Unknow primary tumor	33	27	6	81.8
Total	851	623	228	73.2

Legend: NET = neuroendocrine tumor, PET/CT = positron emission tomography/computed tomography, n = number, pos = positive, neg = negative, GEP = gastro-entero-pancreatic, MEN = multiple endocrine neoplasia, NA = not available, and PRRT = peptide receptor radioligand therapy.

**Table 4:** Scans performed in confirmed GEP NETs, requested by the local oncology ward.

	n		Grade		PET pos	PET neg	Detection Rate
Scans Classified by Primary Tumor Site	n	%		n	n	n	%
Pancreas	239	37.8	G1	81	57	24	70.4
			G2	135	101	34	74.8
			G3	11	8	3	72.7
			NET-NA	12	9	3	75.0
Ileum	326	51.5	G1	134	102	32	76.1
			G2	164	122	42	74.4
			NET-NA	28	24	4	85.7
Pancreas and ileum	1	0.2	G1	1	1	0	100.0
Duodenum	22	3.5	G1	7	5	2	71.4
			G2	15	6	9	40.0
			NET-NA	1	1	0	100.0
Jejunum	9	1.4	G1	7	7	0	100.0
			G2	1	1	0	100.0
Colon	4	0.6	G1	3	2	1	66.7
			G2	1	0	1	0.0
Sigma	2	0.3	G2	1	0	1	0.0
			NET-NA	1	1	0	100.0
Rectum	7	1.1	G2	6	6	0	100.0
			G3	1	1	0	100.0
Gastric	7	1.1	G1	2	1	1	50.0
			G2	5	3	2	60.0
Cecum	5	0.8	G2	5	5	0	100.0
Vater’s papilla	2	0.3	G1	2	1	1	50.0
Appendix	2	0.3	G1	2	0	2	0.0
Gallbladder	1	0.2	G2	1	0	1	0.0
Liver_CUP	1	0.2	G2	1	1	0	100.0
Ileal valve	5	0.8	G1	2	2	0	100.0
			G2	3	1	2	33.3
total	633	100.0		633	468	165	73.9

Legend: NET = neuroendocrine tumor, PET = positron emission tomography, n = number, pos = positive, neg = negative, GEP = gastro-entero-pancreatic, NA = not available, and CUP = tumor of unknown primary.

## Data Availability

Access to the anonymous electronic archive is regulated by the local ethics committee.
